# Report of High-Risk Carbapenem-Resistant *K. pneumoniae* ST307 Clone Producing KPC-2, SHV-106, CTX-M-15, and VEB-1 in Greece

**DOI:** 10.3390/antibiotics14060567

**Published:** 2025-05-31

**Authors:** Maria Chatzidimitriou, Pandora Tsolakidou, Maria Anna Kyriazidi, Sotiris Varlamis, Ilias S. Frydas, Maria Mavridou, Stella Mitka

**Affiliations:** 1 Department of Biomedical Sciences, International Hellenic University, 57400 Thessaloniki, Greece; svarlamis@ihu.gr (S.V.); ifrydas@ihu.gr (I.S.F.); mavridoumaria@ihu.gr (M.M.); mitka@ihu.gr (S.M.); 2 Microbiology Department, Hospital of Volos, Polymeri 134, 38222 Volos, Greece; ptsolakidou@ihu.gr; 3 Medical School, Faculty of Health Sciences, Aristotle University of Thessaloniki, 54124 Thessaloniki, Greece; mkyriaza@auth.gr

**Keywords:** *K. pneumoniae*, carbapenems, Greece, high-risk clone, ST307

## Abstract

Background/Objectives: *Klebsiella pneumoniae* ST307 is emerging as a significant global high-risk antimicrobial-resistant (AMR) clone with a notable capacity to acquire and disseminate resistance genes. However, there is limited research on the pathogenicity, virulence, and adaptation of ST307 strains and on the clinical characteristics of infected patients. Methods: In this study, a carbapenem-resistant *K. pneumoniae (CRKP)* ST307 strain named U989 was isolated from a urine culture of a hospitalized patient in Volos, Greece, in July 2024. Whole-genome sequencing was performed to identify resistance genes to β-lactams *bla*_KPC-2_, *bla*_CTX-M-15_, *bla*_TEM-1B_*, bla*_OXA-1_, *bla*_OXA-10_, *bla*_SHV-106_, and *bla*_VEB-1_ and resistance genes to other antibiotics. Results: A genomic analysis also revealed the presence of virulence factors such as *iut*A, *clp*K1, *fyu*A, *fim*H, *mrk*A, *Irp*2, and *Tra*T and an IncFiB(pQil)/IncFII(K) replicon, which harbors the *bla*_KPC-2_ gene. Additionally, the transposable element Tn4401 was identified as a key vehicle for the mobilization of the *bla*_KPC-2_ resistance gene. Finally, this is the report of a high-risk *CRKP* ST307 clone expressing KPC-2, SHV-106, CTX-M-15, and VEB-1 *bla* genes in Greece. Conclusions: The coexistence of these resistance genes in addition to aminoglycoside, quinolone, and other resistance genes results in difficult-to-treat infections caused by respective carrier strains, often requiring the use of last-resort antibiotics and contributing to the global challenge of antimicrobial resistance.

## 1. Introduction

The global rise of antibiotic-resistant bacteria poses a significant threat to public health, with *Klebsiella pneumoniae* emerging as one of the most concerning pathogens [1]. This pathogen is a Gram-negative, non-motile, encapsulated, and rod-shaped bacterium belonging to the *Enterobacteriaceae* family and can lead to several pathogenic conditions, including pneumonia, septicemia, endocarditis, and pyogenic liver abscesses [2,3]. It is commonly found in healthcare settings and has developed a resistance to multiple classes of antibiotics, thus, increasing the complication level of treatment, which results in high morbidity and mortality rates in immunocompromised patients and in developed nations with bad hygiene conditions in hospitals [4,5]. Worldwide, the majority of carbapenem-resistant *K. pneumoniae (CRKP)* strains belong to the notorious CC258 clone, including ST258, ST11, ST340, ST437, and ST512 [6].

In Greece, one of the highest carbapenem resistance rates was reported in Gram-negative bacteria globally, with an increase of 30% in hospital wards and 60% in intensive care units (ICUs) between 2001 and 2008 in carbapenem prevalence [7]. Another study in the ICUs of three university hospitals in Athens, in 2002, recovered 17 *K. pneumoniae* isolates harboring *bla*_VIM-1_ over a 3-month period, and at least 12 isolates were clinically relevant [8]. In addition, a study performed in 21 Greek hospitals showed that by the end of 2008 there was a strain replacement from *bla*_VIM-1_ to *bla*_KPC-2_, and 96% of *K. pneumoniae* isolates were of the ST258 lineage [9]. Moreover, another study showed that other ST types carrying *bla*_KPC_ circulate among the almost 40% of *K. pneumoniae-*harboring *bla*_KPC_ in Greece [10]. Furthermore, a study with 165 patients performed between 2016 and 2019 assessed the molecular epidemiology of CPKP isolates and found 128 pulsotypes and 17 clusters, indicating the extended dissemination of different clinical isolates in hospital ICUs and that 74% of them harbored KPC genes [11]. Finally, the most recent study in Greece showed a high number of within-hospital transmission events in 15 hospitals involving international high-risk clones, such as ST258/512 and ST11, and the Greek endemic high-risk clones ST39 and ST323 [12]. Thus, continuous national molecular surveillance is essential to track emerging antimicrobial resistance threats and to assess the effectiveness of the applied control measures.

Among the various annotated genomes of *K. pneumoniae*, ST307 has gained attention due to its widespread dissemination and its association with multidrug resistance, particularly the production of extended spectrum beta-lactamases and carbapenemases, like KPC-2 and OXA-48-like carbapenemases. Initially reported in 2013, ST307 has since been identified and disseminated worldwide, and it is often linked to severe hospital outbreaks [13,14,15,16,17]. However, there is limited research concerning the pathogenicity, virulence, and fitness of ST307, thus making it difficult to fully understand the mechanisms driving its global spread.

In this retrospective study we aimed to elucidate the molecular characteristics of antibiotic resistance genes, virulence factors, and various mobile genetic elements linked to the *K. pneumoniae* U989 isolate of ST307. The latter strain is a multi-resistant isolate from a urine culture of a male patient who was hospitalized in the Urological Department of Volos General Hospital in Greece. This study investigates the genetic components associated with the *bla*_KPC-2_ gene, which is a major contributor to carbapenem resistance. The analysis identified a plasmid replicon named IncFiB(pQil)/IncFII(K), which harbors the *bla*_KPC-2_ gene [18]. This is the first time that a *bla*_KPC-2_ plasmid IncFiB(pQil)/IncFII(K) was isolated from ST307 *K. pneumoniae*. Additionally, it was shown that the identified transposable element Tn4401 plays a crucial role in the mobilization of this resistance gene.

## 2. Materials and Methods

The *K. pneumoniae* U989 isolate was obtained from a urine culture of a 70-year-old male patient hospitalized at the General Hospital of Volos, Greece, between 15 July and 17 July 2024. The patient had a lower urinary tract infection following urodynamic testing, with a history of chronic obstructive voiding, vesicoureteral efflux (VUR), and urethral stricture. He was scheduled for a transurethral prostatectomy. Ethical approval was not required for this study, as the isolate was collected during routine clinical diagnostics.

Urine sampling was performed in the microbiology department of the General Hospital of Volos and cultured in routine media. Identification and antimicrobial susceptibility testing was conducted using the Vitek-2 automated system (Biomerieux, Marcy-l’Étoile, France) [19]. Susceptibility testing for ceftazidime–avibactam (CAZ-AVI) combination was performed using minimal inhibitory concentration (MIC) gradient E-tests (Liofilchem Roseto degli Abruzzi, Italy) [20]. The determination of the minimal inhibitory concentration was performed according to European Committee on Antimicrobial Susceptibility Testing (EUCAST) guidelines (https://www.eucast.org/clinical_breakpoints (accessed on 15 July 2024).

Susceptibility to colistin was tested using a cation adjusted Mueller–Hinton broth (CAMHB) microdilution method (Liofilchem), and tigecycline susceptibility was evaluated using susceptibility breakpoints (susceptible <=2 mcg/mL, intermediate 4 mcg/mL, resistant >=8 mcg/mL), which were approved by the US Food and Drug Administration [21]. Carbapenem resistance was set as the limited microbe threshold exhibiting resistance to any of the tested carbapenems. The isolate was phenotypically tested for metallo-beta-lactamase (MBL) and *K. pneumoniae* carbapenemase (KPC) production using ethylenediaminetetraacetic acid (EDTA) and phenylboronic acid (PBA) [22].

A multiplex lateral flow immunoassay (LFIA; NG-test CARBA 5; NG Biotech, Guipry-Messac, France) was used to detect common carbapenem resistance genes in a single reaction, including *bla*_KPC_, *bla*_NDM_, *bla*_IMP_, and *bla*_OXA-48_-like genes [23]. Detection limits of purified enzymes for New Delhi MBL (NDM), KPC, active-on-imipenem (IMP), Verona integron-mediated MBL (VIM), and oxacillinase (OXA-48-like) were 150 pg/mL, 600 pg/mL, 200 pg/mL, 300 pg/mL, and 300 pg/mL, respectively.

For the tested isolate a blood culture identification panel (Biofire, Biomerieux, Marcy-l’Étoile, France) was used to identify common carbapenem resistance genes in the isolate tested, such as *bla*_KPC_, *bla* _NDM_, bla_VIM_, and *bla*_OXA_-48-like genes [24].

Genomic DNA was extracted from overnight bacterial culture using the PureLink Genomic DNA Mini Kit (Invitrogen, Carlsbad, CA, USA), according to the manufacturer’s protocol for Gram-negative bacteria.

NGS was performed in a private laboratory in Greece. Libraries were prepared using Ion Torrent technology and the Ion Chef Flow Diagram (Thermo Fisher Scientific, Waltham, MA, USA). DNA libraries were sequenced using the 5SXLS system, and raw sequences were analyzed using the Ion Torrent Suite v.s1010 (Thermo Fisher Scientific). The online Galaxy Server tool (https://usegalaxy.org/, accessed on 15 July 2024) and the Center for Genomic Epidemiology (https://www.genomicepidemiology.org/, accessed on 15 July 2024) database were then used to assess and annotate analyzed sequences. Reads quality was estimated using the FastQC tool (Galaxy version 0.75) and was improved using FASTQ Quality Trimmer (Galaxy version 1.1.5). The bacterial genome was assembled using the online Create Assemblies tool with the Unicycler pipeline (Galaxy version 0.5.0). Resistance genes were identified using ABRIcate for the mass screening of antimicrobial and virulence genes (Galaxy Version 1.0.1), and replicons were detected using Plasmid Finder (Galaxy Version 2.1.6), which enabled plasmid replicon typing. Gene detection was based on ≥90% identity and at ≥60% coverage thresholds. The K locus and O serotype were designated with the use of Kaptive ((https://kaptive-web.erc.monash.edu/) accessed on 15 July 2024). Finally, integrative conjugative elements (ICEs) were detected using ICE finder ((https://bioinfo-mml.sjtu.edu.cn/ICEfinder accessed on 15 July 2024).

## 3. Results

The U989 isolate exhibited resistance to most of the antibiotic classes, including β-lactams (penicillins, cephalosporins, and monobactams), fluoroquinolones, aminoglycosides (excluding gentamicin), tetracyclines, and folate pathway inhibitors. It remained susceptible to colistin, gentamicin, ceftazidime–avibactam, and imipenem and intermediately resistant to meropenem and amikacin (Supplementary Materials Table S1).

The patient was treated with a ceftazidime–avibactam monotherapy and showed a clinical improvement without the recurrence of the infection.

Whole-genome sequencing (WGS) was performed as part of a targeted investigation due to the isolate’s multidrug-resistant profile and intermediate susceptibility to meropenem.

The whole-genome sequencing (WGS) of *K. pneumoniae* U989 showed a total length of 5,757,733 base pairs (bp) distributed across 91 contigs. The largest contig was 513,378 bp. The N50 and N90 values were 180,980 bp and 63,649 bp, respectively, showing a moderately contiguous assembly. The L50 and L90 values were 11 and 32, respectively. The area under the Nx curve (auN) was measured at 201,997 bp, and the GC content of the genome was 57.02% (Supplementary Materials Table S2). The isolate was designated to the K102 capsular type and O2afg serotype.

The genetic makeup and resistance genes of the *K. pneumoniae* U989 isolate are shown in Table 1.

The isolate genome analysis revealed the presence of multiple beta-lactamase genes, including *bla*_KPC-2_, *bla*_CTX-M-15_*, bla*_TEM-1B_, *bla*_OXA-1_, *bla*_OXA-10_, *bla*_SHV-106_, and *bla*_VEB-1_. The *bla*_KPC-2_ gene was carried by the IncFIB(pQil)/IncFII(K) replicon on transposon Tn4401 [18]. Other identified replicons were ColRNAI and INcA/C. The association of *K. pneumoniae* U989 with aminoglycoside and quinolone resistance genes, such as *ant*(2″)-Ia, *aad*A1, *aph*(6)-Id, *aac(*3)-IIa, *aac*(6′)-Ib-cr, and *rmt*B, along with other resistance determinants, like *Oqx*A, *Oqx*B, *ARR*-2, *sul*2, *dfr*A14, *cat*B3, *fos*A, *cml*A1, and *tet*(G), underscores its capacity to resist multiple classes of antibiotics [25,26,27].

The presence of virulence factors in *K. pneumoniae* U989, such as *iut*A, *clp*K1, *fyu*A, *fim*H, *mrk*A, and *Irp*2, along with the presence of *Tra*T in ST307 strains enhances their ability to cause severe infections and resist host immune defenses (Table 2).

Five ICEs were detected in the study isolate (Supplementary Materials Figure S1).

The ICEs carried genes for the Type 4 secretion system which facilitate horizontal gene transfer. The presence of ICEKp1 enhances the pathogenic potential of *K. pneumoniae* and contributes to its adaptability and persistence in various environments [20]. In this study, both the *fyu*A gene for the synthesis of the receptor for yersiniabactin and the *irp*2 yersiniabactin biosynthetic gene were carried by an integrative conjugative element ICEEcoED1a-1, which belongs to the ICEKp1 family.

The analysis identified multiple insertion sequences and replicons associated with *K. pneumoniae*, including IS6100, ISKpn14, ISKpn25, and others from the IS3, IS6, and IS5 families (Table 3). Replicons from the incompatibility groups F, ColE, and C/A2 were also identified, with group F being the most common in *Enterobacteria*. Notably, IncFIB(K) plasmids, which are linked to high-risk multidrug-resistant clones, like ST258 and ST307, contain mobile genetic elements from the IS3 and IS5 families [18]. These elements, including composite transposons like cn_50683_ISEcl1, play a key role in gene transfer and in the spread of antibiotic resistance and virulence genes in bacteria.

Furthermore, the analysis revealed the insertion sequences IS6100, ISKpn14, ISKpn25, IS6100, IS102, IS903, ISEcl1, cn_50683_ISEcl1, ISEc15, ISkpn1, and IS5075 (Table 3), and it is already known that the IS3 family is frequently associated with *K. pneumoniae* [28].

Replicons from the incompatibility group F, ColE and C/A2, were revealed from the analysis of the strain. Group F is a more frequent incompatibility group in *Enterobacteria* [29]. The IncFIB(pQil)/IncFII(K) replicon harbored the *bla*_KPC-2_ gene.

The mobile genetic elements identified in Table 3, including insertion sequences, transposons, integrative conjugative elements, and plasmid replicons, play a fundamental role in shaping the resistome and virulome of *K. pneumoniae* isolates by mediating the horizontal gene transfer of antibiotic resistance and virulence determinants. Insertion sequences such as IS6100, ISKpn14, ISKpn25, and IS903 are related to mgrB gene mutations [30]. Plasmid replicons from incompatibility groups such as IncFIB, IncFII, IncA/C, and ColRNAI support the maintenance and propagation of large multidrug resistance plasmids across diverse Enterobacteriaceae [31]. Tn4401 is a known vehicle for the dissemination of carbapenemase genes such as blaKPC-2 [32].

## 4. Discussion

The global emergence and spread of the *K. pneumoniae* ST307 lineage, particularly in association with KPC-2 gene expression, have been documented across various regions outside Greece. In Texas, Castanheira et al. (2013) highlighted the rapid clonal expansion of ST307 in two hospitals, marking its aggressive presence alongside the ST258 lineage [13]. Similarly, Bonura et al. (2015) observed the rise of ST307 among other non-ST258 clones in Sicily, contributing to the spread of *bla*_KPC_-carrying *K. pneumoniae* [15]. In Korea, Park et al. (2015) identified ST307 as a significant genotype among ciprofloxacin-resistant strains, emphasizing its resistance profile [33]. In Tunisia, it was reported that the ST307s are involved in disseminating multidrug-resistant *K. pneumoniae*, particularly with the blaCTX-M-15/IncFIIk plasmids, and in Italy, the diversity, virulence, and antimicrobial resistance of the ST307 clone was further investigated, underscoring its clinical significance as a KPC producer [25,34]. A comprehensive review by Wyres et al. (2019) documented the global dissemination of the CTX-M-15-associated ST307 strain, highlighting its role in the antibiotic resistance crisis [26]. Finally, in China the emergence of ST307 co-producing CTX-M, SHV, and KPC in pediatric patients was reported, indicating the strain’s ongoing global impact [27]. The ST307 K. pneumoniae lineage comprises a variety of additional resistance and virulence determinants, integrative conjugative elements, and phages [25,26,27,35,36].

This study focuses on *a K. pneumoniae* ST307 isolate harboring multiple β-lactamase genes, including *bla*_KPC-2_, *bla*_CTX-M-15_*, bla*_TEM-1B_, *bla*_OXA-1_, *bla*_OXA-10_, *bla*_SHV-106_, and *bla*_VEB-1_. The presence of these genes indicates that there is a high level of resistance to various classes of beta-lactam antibiotics, thus complicating treatment options. The *bla*_KPC-2_ gene confers a resistance to carbapenems, and this is often considered the last line of defense against Gram-negative infections [37]. The association of the *K. pneumoniae* U989 isolate with aminoglycoside and quinolone resistance genes, such as *ant*(2″)-Ia, *aad*A1, *aph*(6)-Id, *aac(*3)-IIa, *aac*(6′)-Ib-cr, and *rmt*B, along with other resistance determinants, like *Oqx*A, *Oqx*B, *ARR*-2, *sul*2, *dfr*A14, *cat*B3, *fos*A, *cml*A1, and *tet*(G), underscores its capacity to resist multiple classes of antibiotics. This multifaceted resistance is primarily due to the acquisition of various genes that encode for mechanisms like drug modification, ribosomal protection, and active efflux, which collectively enable *K. pneumoniae* to evade the effects of aminoglycosides, fluoroquinolones, and other critical antibiotics (Table 1). The presence of *aac(6′)-Ib-cr*, a gene that not only modifies aminoglycosides but also confers a resistance to fluoroquinolones, illustrates the complex interplay of resistance mechanisms [30]. Additionally, *rmt*B contributes to the high-level aminoglycoside resistance through ribosomal methylation [38]. The point mutations in efflux pump genes *Oqx*A, *Oqx*B, *gyr*A, and *par*C further enhance fluoroquinolone resistance. The combination of these resistance mechanisms, especially in high-risk clones like ST307, complicates treatment options, often necessitating the use of last-resort antibiotics and exacerbating the global challenge of antimicrobial resistance (Table S2) [14]. The coexistence of the blaKPC-2, blaVEB-1, blaCTX-M-15, and blaSHV-106 genes in the ST307 U989 isolate contributes to an extensive β-lactam resistance profile by simultaneously targeting different subclasses of β-lactam antibiotics, including carbapenems, cephalosporins, penicillins, and monobactams. The blaKPC-2 gene encodes a class A carbapenemase that hydrolyzes carbapenems and many other β-lactams, while blaVEB-1 encodes an extended-spectrum β-lactamase capable of inactivating third-generation cephalosporins and aztreonam.

The *bla*CTX-M-15 gene is among the most widespread ESBLs globally and is particularly effective against cefotaxime and ceftazidime. Although less commonly studied, *bla*SHV-106 has been associated with an enhanced resistance to penicillins and cephalosporins, and its co-expression with other β-lactamases may amplify the overall resistance phenotype. The convergence of these genes likely provides substrate redundancy and synergistic enzymatic activity, thereby reinforcing the β-lactam resistance phenotype and reducing the efficacy of most β-lactam-based treatments [38,39]. This genetic background explains the limited therapeutic options and the observed susceptibility only to last-resort combinations, such as ceftazidime–avibactam. Moreover, the presence of *aac(6′)-Ib-cr* in this isolate confers a dual resistance by enzymatically modifying both aminoglycosides and fluoroquinolones, which further complicates treatment. Specifically, *aac(6′)-Ib-cr* acetylates aminoglycosides, like tobramycin and amikacin, concurrently modify fluoroquinolones, impairing their interaction with target enzymes, such as DNA gyrase and topoisomerase IV. In combination with chromosomal mutations in *gyrA* (S83I) and *parC (*S80I), this gene promotes a high-level fluoroquinolone resistance [40,41]. Additionally, recent studies suggest the possibility of epistatic interactions among co-located resistance genes, particularly on plasmids of the IncF family, which may influence gene expression and the phenotypic impact of resistance determinants [31,42]. The simultaneous presence of *bla*CTX-M-15 and *aac(6′)-Ib-cr* on such plasmids may facilitate a co-selection under sub-inhibitory antibiotic exposure, contributing to the stable maintenance and dissemination of multidrug resistance. These epistatic effects represent a potential evolutionary advantage, supporting the clonal expansion of high-risk lineages like ST307 and underlining the urgent need for genomic surveillance and novel treatment approaches. The resistance gene profile of our tested isolate was compared to published ST307 genomes from Italy, Tunisia, Germany, and China and showed a conserved core of multidrug resistance and virulence genes.

The association of *K. pneumoniae* with aminoglycoside and quinolone resistance genes, such as *ant(*2″)-Ia, *aad*A1, *aph*(6)-Id, *aac*(3)-IIa, *aac*(6′)-Ib-cr, and *rmt*B, as well as other resistance genes, like *ARR*-2, *sul*2, *dfrA*14, *cat*B3, *fos*A, *cml*A1, and *tet*(G), is indicative of its capacity to resist multiple classes of antibiotics, making it a significant challenge in clinical settings.

Concerning meropenem susceptibility, one plausible explanation for this phenotype is the weak expression of the *bla*KPC-2 gene, resulting in the reduced production of the KPC-2. Mutations in the promoter region of the *bla*KPC-2 gene can result in a reduced enzyme production, potentially leading to the underestimation of resistance in diagnostic settings [37,38,40]. As no gene expression quantification data were available, the *bla*_KPC-2_ expression level was only assessed based on the phenotype, and a further expression analysis is needed.

ST307 *Klebsiella pneumoniae* is a globally high-risk clone known for its notable capacity to acquire and disseminate resistance genes and its association to multidrug resistance and various virulence factors that contribute to the pathogenicity and survival in hostile environments [14,27]. The coexistence of β-lactamase genes within a single strain underscores the importance of vigilant surveillance, appropriate antimicrobial stewardship, and the development of new therapeutic strategies to combat the spread of such highly resistant organisms. Additionally, the common expression of aminoglycoside, quinolone, and other resistance genes makes infections caused by such strains difficult to treat, often requiring the use of last-resort antibiotics and contributing to the global challenge of antimicrobial resistance. These findings reinforce the role of ST307 as an emerging high-risk clone in southern Europe.

The comparison of the ST307 U989 isolate with other *K. pneumoniae* sequence types recovered from the same hospital revealed the co-circulation of several high-risk clones, including ST15 producing both *bla*NDM-1 and blaVIM-1 with a pandrug-resistant profile, ST11 strains harboring *bla*NDM-1 alone or in combination with *bla*OXA-48 with varying susceptibility to colistin and trimethoprim–sulfamethoxazole, and ST39 carrying both KPC-2 and VIM-1 along with multiple *bla*SHV-type beta-lactamases, all of which were associated with diverse replicon backgrounds, including IncFIB(K), IncFIA, IncC, and IncR, and exhibited the presence of mobile genetic elements such as Tn4401 and insertion sequences, indicating a high degree of genomic plasticity that facilitates horizontal gene transfer and the adaptation to the hospital environment (Table 4) [43,44,45,46].

In addition, this study has some limitations. Additional experiments and analyses are required, such as conjugation experiments to assess the horizontal transfer potential of the resistance plasmids and to further elucidate the mechanisms underlying the observed low *bla*_KPC_ expression. Moreover, further research is needed to confirm these findings and further identify the precise causative factors. Also, the isolate was obtained from a single patient in a tertiary hospital setting, raising the possibility of selection bias and limiting the generalization of our findings. Finally, clinical implications due to the use of the imipenem monotherapy in phenotypically sensitive isolates in complicated urinary infections remain uncertain and require further investigation.

## 5. Conclusions

The isolation of CRKP ST307 along with the coexistence of resistance and virulence genes highlight the adaptability and resilience of the isolated *K. pneumoniae* U989 clone, making it a significant public health threat, especially in healthcare settings where antibiotic resistance is a major challenge. The emergence of ST307 strains carrying resistance genes underscores the importance of continuous surveillance, effective infection control measures, and the development of novel antibiotics to combat these antibiotic-resistant pathogens. Our findings should influence clinical practice and infection control. The intermediate susceptibility to meropenem, despite the presence of *bla*_KPC-2_ in the *K. pneumoniae* U989 isolate, raises concerns about the reliance on routine phenotypic methods. This could lead to the underestimation of resistance and inappropriate infection control. Thus, this case highlights the necessity of integrating next-generation sequencing methodologies into routine hospital microbiology for unusual resistance profiles. More specifically we recommend the following: (1) to perform molecular typing of all CRKP isolates, (2) to assess the interpretation of imipenem susceptibility when *bla*_KPC-2_ is detected, and (3) to perform a multicenter genomic screening for the ST307 prevalence in Greece.

## Figures and Tables

**Table 1:** Genetic makeup and resistance genes of *K. pneumoniae* U989 isolate.

Resistance Type	Origin	Resistance Genes
Beta-lactamases	Plasmid-mediated	*bla*_KPC-2_, *bla* CTX-M-15, *bla* TEM-1B, *bla* SHV-106, *bla* OXA-1, *bla* OXA-10, and *bla* VEB-1
Beta-lactamases	Point mutations	*OmpK35* and *ompK36*
Aminoglycosides	Plasmid-mediated	*ant*(2″)-Ia, *aad*A1, *aph*(6)-Id, *aac(*3)-IIa, *aac*(6′)Ib-cr, and *rmt*B
Fluoroquinolones	Plasmid-mediated	*aac*(6′)Ib-cr and *oqxA*, *oqxB*
Fluoroquinolones	Point mutation	*parC*_S80I and *gyrA*_S83I,
Rifampicine, Chloramphenicol, Sulfamethoxazole, Fosfomycin	Plasmid-mediated	*arr*-2, *sul*2, *dfr*A14, *cat*B3, *fos*A, *cml*A1, and *tet(*G)
Quaternary ammonium compound efflux	Plasmid-mediated	qacEdelta1

**Table 2:** Virulence factors of *K. pneumoniae* U989 strain.

Virulence Factor	Function
*iutA*	Iron uptake (siderophore receptor)
*clpK*1	Heat-shock protein, associated with survival in hostile environments
*fyuA*	Iron acquisition and transport, related to pathogenicity
*fimH*	Adhesion to host cells, critical for colonization
*mrkA*	Type 3 fimbriae, involved in biofilm formation and attachment to surfaces
*Irp*2	Iron receptor molecule

**Table 3:** Mobile genetic elements of *K. pneumoniae* U989 isolate.

Category	Details
**Insertion Sequences Identified**	IS6100, ISKpn14, ISKpn25, IS102, IS903, ISEcl1, cn_50683_ISEcl1, ISEc15, ISkpn1, and IS5075
**Associated Families**	IS3, IS6, IS5, and IS110
**Replicons Identified**	IncFIB(pQil)/IncFII(K), IncFIB(K), ColRNAl, and InC/A2
**Key Plasmid**	IncFIB(K) associated with high-risk, multidrug-resistant clones (e.g., ST258 and ST307)
**Mobile Genetic Elements**	Composite transposons (e.g., cn_50683_ISEcl1),
**Insertion Sequence Family and Group**	IS3 family, Group IS2 (ISEcl1, composite transposon cn_50683_ISEcl1
	IS5 family, Group IS903 (IS102 and IS903)
	IS3 family, Group IS51 (ISEc15)
	IS3 family, Group IS150 (ISkpn1)
	IS110 family, Group IS1111 (IS5075)
**Transposons Identified**	Tn4401, Tn5403
**Clinical Relevance**	High-risk *K. pneumoniae* clones (ST307) have both virulence factors and multidrug resistance, making infections difficult to treat

**Table 4:** Comparative analysis of *K. pneumoniae* high-risk clones from Volos Hospital.

Feature	ST307 (U989)	ST15 (A436)	ST11 (A165)	ST11 (838Gr)	ST39 (A165)
**Specimen**	Urine	Blood	Blood	Urine	Blood
**Carbapenemases**	*bla*KPC-2	*bla*NDM-1, *bla*VIM-1	*bla*NDM-1, *bla*OXA-48	*bla*NDM-1,	*bla*KPC-2, *bla*VIM-1
**Other β-lactamases**	*bla*CTX-M-15, *bla*SHV-106, *bla*VEB-1, *bla* TEM-1B, *bla*OXA-1, *bla*OXA-10	*bla*CTX-M-15, *bla* SHV-28, *bla*TEM-1, *bla*OXA-1	*bla*CTX-M-14b, *bla*SHV-182	*bla*CTX-M-15, *bla*TEM-1B, *bla*VEB-1, *bla*SHV-11, *bla*OXA-10	*bla*SHV-40, *bla*SHV-56, *bla*SHV-79, *bla*SHV-85, *bla*SHV-89
**Resistance profile**	XDR, susceptible to colistin, gentamicin, CAZ-AVI	PDR	Susceptible to gentamicin, colistin, TMP-SMX	Colistin-resistant MDR	Colistin sensitive
**Replicons**	IncFIB(pQil)/FII(K), IncA/C, ColRNAI	IncA/C2, IncFIB(K), IncFIA(HI1) IncFII(K)	IncFIB(K), IncFIA(HI1), IncFII(K), IncR, Col440II	IncFIA, IncC, IncR, repB	ColRNAI, IncC, IncFIB(K), IncFIB(pQil), IncFII(K)
**Virulence genes**	*iutA*, *fyuA*, *mrkA*, *fimH*, *clpK1*, *irp2*, *traT*	*mrk*, *fim*, *yersiniabactin* cluster	*irp1*, *irp2*, *ybtE*, *iutA*, *ompA*, *rcsA/B*	*entA-S*, *iroN*, *fyuA*, *iutA*, *T6SS*	Not included
**Capsular type/O-antigen**	KL102/O2afg	KL48	KL24/O2a	KL24/O2a	KL23/O2afg
**Mobile genetic elements**	Tn4401, ICEKp1, IS6/IS5/IS3 families	Class 1 integrons, multiple IS elements	9 insertion sequences,	Tn5403, 3 integrons, ICEs	ISs, phage regions, CRISPRs, ompK mutations
**Year of Isolation**	2024	2024	2024	2024	2020

## Data Availability

The whole genome of *K. pneumoniae* was deposited in DDBJ/ENA/Genbank under the accession number JBGIYZ000000000.

## Supplementary Materials

The following supporting information can be downloaded at: https://www.mdpi.com/article/10.3390/antibiotics14060567/s1, Table S1: Sensitivity of *K. pneumoniae* U989 isolate to antibiotics.; Table S2: Whole-genome sequencing (WGS) assembly statistics for *K. pneumoniae* U989 isolate. Figure S1: Integrative conjugative elements of *K. pneumoniae* U989 isolate.
