# Supplementary material for: Report of High-Risk Carbapenem-Resistant K. pneumoniae ST307 Clone Producing KPC-2, SHV-106, CTX-M-15, and VEB-1 in Greece
Source: Antibiotics (Basel). 2025 May 31;14(6):567. doi: 10.3390/antibiotics14060567 (PMC12189958; doi:10.3390/antibiotics14060567)
Supplement: Supplementary file 1 [file antibiotics-14-00567-s001.zip › antibiotics-3576178-Supplementary Materials.pdf]

antibiotics

MDPI

Supplementary Materials

Table S1. Sensitivity of *K. pneumoniae* U989 isolate to antibiotics.

Antibiotic	MIC mg/L	Interpretation
Ampicillin	≥ 32	R
Amoxycillin-clavulanic acid	≥ 32	R
Ampicillin-sulbactam	≥ 32	R
Ticarcillin-clavulanic acid	≥ 128	R
Piperacillin	≥ 128	R
Piperacillin-tazobactam	≥ 128	R
Cefuroxime	≥ 64	R
Cefepime	≥ 64	R
Cefotaxime	≥ 64	R
Ceftazidime	≥ 64	R
Aztreonam	≥ 64	R
Imipenem	≤ 0.25	S
Meropenem	8	I
Amikacin	8	I
Gentamicin	≤ 1	S
Tobramycin	≥ 16	R
Ciprofloxacin	≥ 4	R
Levofloxacin	≥ 8	R
Moxifloxacin	≥ 8	R
Ofloxacin	≥ 8	R
Tigecycline	≥ 8	R
Chloramphenicol	≥ 64	R
Colistin	2	S
Trimethoprim-sulfamethoxazole	≥ 320	R
Ceftazidime-avibactam	1	S

 https://doi.org/10.3390/antibiotics14060567

Table S2. Whole-genome sequencing (WGS) assembly statistics for *K. pneumoniae* U989 isolate.

Parameter	Value
Number of contigs	91
Number of contigs (≥ 0 bp)	116
Number of contigs (≥ 1000 bp)	80
Largest contig (bp)	513,378
Total length (bp)	5,757,733
Total length (≥ 0 bp) (bp)	5,765,284
Total length (≥ 1000 bp) (bp)	5,749,833
N50 (bp)	180,98
N90 (bp)	63,649
auN (bp)	201,997
L50	11
L90	32
GC content (%)	57.02

Legend: *N50*: The length of the contig for which all contigs of that length or longer cover 50% of the genome. *N90*: The length of the contig for which all contigs of that length or longer cover 90% of the genome. *L50*: The minimum number of contigs whose cumulative length makes up 50% of the total assembly size. *L90*: The minimum number of contigs whose cumulative length makes up 90% of the total assembly size. *auN*: Area under the Nx curve; a weighted measure of contiguity across all values of N.

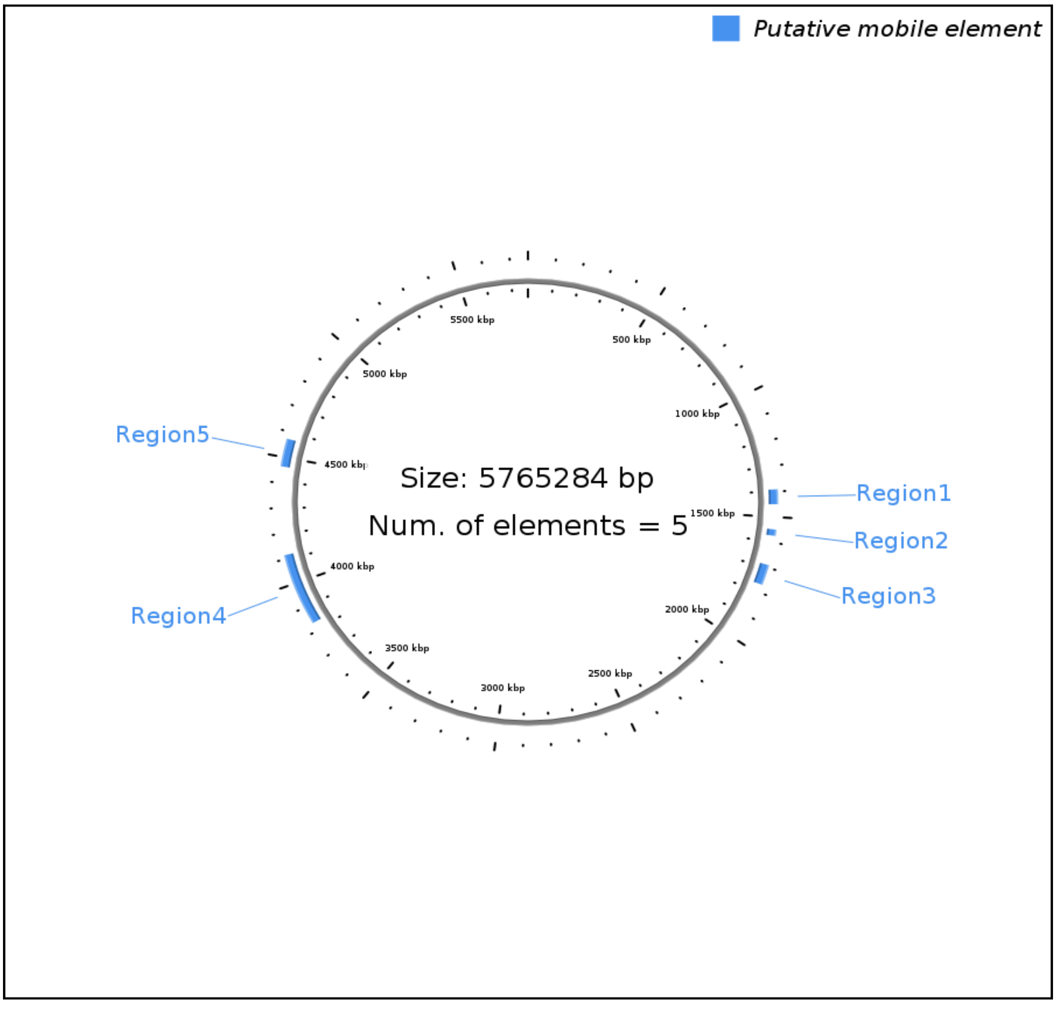

Figure S1. Integrative conjugative elements of *K. pneumoniae* U989 isolate.
